# Efficacy of Back Bracing in Treating Chronic Low Back Pain

**DOI:** 10.3390/brainsci14111100

**Published:** 2024-10-30

**Authors:** John S. Vick, Jessica Zimmerman, Stephanie Hicks, Abigail Biekert, Alaa Abd-Elsayed

**Affiliations:** 1 Associated Physicians Group, St. Louis, MO 63141, USA; 2 Department of Anesthesiology, University of Wisconsin-Madison, Madison, WI 53706, USA

**Keywords:** low back pain, chronic pain, back bracing, orthoses

## Abstract

Chronic low back pain (CLBP) negatively impacts quality of life and contributes to a significant economic burden. One conservative management strategy for CLBP is lumbar back bracing. Despite the benefits of back bracing for improving pain and function, there remains hesitance to use the therapy long term due to unfounded fear related to muscle weakness, deconditioning, or joint contracture. Objective: The purpose of this study was to examine the outcomes for patients with CLBP who were managed with lumbar back bracing and physical therapy. Methods: This was a single-site, retrospective chart review. Results: Patients were included in the study if they were treated for CLBP with back bracing for at least one hour daily and physical therapy for twelve weeks. Pain was assessed at three, six, and twelve months using the 11-point Visual Analogue Scale (VAS). Function was assessed at three months using the Oswestry Disability Index (ODI). The VAS score reduced from 6.28 +/− 2.32 to 3.96 +/− 2.66 at three months (*p* < 0.001) for 198 patients. At six and twelve months, the VAS score reduced to 3.74 +/− 2.73 (*p* < 0.001) and 3.23 +/− 2.29 (*p* < 0.001), respectively. The total ODI score for 199 patients improved from 46.56 +/− 15.30 to 33.13 +/− 19.99 (*p* < 0.001) at three months. Conclusion: Back bracing in combination with physical therapy is effective for treating low back pain.

## 1. Introduction

Over 20% of Americans experience chronic pain, with over 40% of cases being attributed to back pain [1]. It has been reported that, in a typical week, at least one low back pain patient is seen by almost every primary care provider [2]. Up to a quarter of acute low back pain cases transform into a chronic ailment [3]. Chronic low back pain (CLBP) negatively impacts a person’s quality of life by reducing their ability to work, participate in activities, and overall physical functions [4]. CLBP not only affects individuals but also their family and friends [5]. Total costs attributed to CLBP have been estimated to range between USD 100 billion and USD 200 billion each year [6].

There are several risk factors for developing CLBP. Studies have shown that more females than males experience CLBP [7]. Additionally, the trends indicate that prevalence increases with age. A sedentary lifestyle, including extended time spent sitting or driving, is another risk factor for CLBP [8]. While a sedentary lifestyle can contribute to the development of CLBP, an overly active lifestyle can do the same. More specifically, overexertion while lifting, pushing, or pulling poses a risk [9]. Comorbidities of depression, somatization disorder, and associated poor mental health increase the likelihood of acute low back pain becoming chronic [10]. Excessive pain avoidance can also contribute to chronification.

Over 90% of CLBP cases are nonspecific, meaning the source of pain cannot be identified [3]. For cases where a pain source can be determined, common etiologies include infections, disc herniation, spinal stenosis, tumors, osteoporosis, muscle/tissue damage, and nerve damage [11]. CLBP can be classified as neuropathic, where a nerve is injured, resulting in pain that can radiate down to the buttocks and legs; nociceptive, where muscles or tissues are damaged; or a combination of both [12,13]. It is worth noting that neuropathic CLBP is typically accompanied by worse outcomes and greater comorbidities.

Current treatment options include pharmacological interventions (e.g., acetaminophen and non-steroidal anti-inflammatory drugs [NSAIDs] for nociceptive symptoms and gabapentin for neuropathic symptoms), topical agents such as lidocaine, heat or cold application, massage therapy, acupuncture, chiropractic therapy, and physical therapy [14,15,16,17,18,19,20,21,22,23,24,25,26,27,28,29,30].

Cortical changes in the brain are also associated with chronic low back pain. There is evidence indicating that a reorganization of trunk muscle representation occurs at the motor cortex, therefore leading to postural control deficits and disrupted motor control of key segmental stabilization muscles of the spine [31,32].

Another conservative management modality for CLBP is non-rigid lumbar back bracing (i.e., lumbosacral orthosis [LSO]). Non-rigid LSOs have been shown to improve both pain and function [33,34,35] by stabilizing and supporting the back by limiting, correcting, assisting, or eliminating harmful movements [16]. Back braces also serve as a proprioceptive reminder for patients to improve their dynamic posture and balance [34].

However, there is hesitance to use back braces long term as clinicians often present an unfounded fear related to muscle weakness, deconditioning, or joint contracture [17,33]. A clinical survey (Alberta, Canada) showed that approximately 50% of clinicians (MDs, DCs, and PTs) believe that non-rigid back braces cause muscle atrophy [33]. This result suggests a potential gap in knowledge, which leads to varied clinical practice with respect to brace prescription [33]. Furthermore, many recent studies have demonstrated that non-rigid back braces have no negative effects on trunk muscle function or composition [16,17,36]. Although the effects of rigid versus non-rigid bracing are quite well-known in the extremities, it is possible that clinicians transpose the unwanted effects of rigid extremity bracing to all bracing applications [33].

Despite the publication of several positive clinical outcome studies, a number of clinical practice guidelines for nonspecific low back pain do not recommend the use of back bracing [35]. Conversely, the U.S. Dept. of Health and Human Services Pain Management Best Practices does recommend back bracing for CLBP. Given the aforementioned information, back brace prescription is not consistent or systematic by all physicians or physiotherapists.

The purpose of this study was to examine the outcomes for patients with CLBP who were managed with lumbar back bracing and physical therapy.

## 2. Materials and Methods

### 2.1. Study Design and Patient Selection

This was a single-site, retrospective chart review that received IRB exemption from the WCG IRB. This study included patients from 1 January 2021 to 30 June 2023 with complete follow-up data points. We reviewed 698 patient charts and excluded 499 due to a lack of complete follow-up data. Data were obtained from electronic medical records. Patients were included in this study if they were treated for CLBP using a back brace (Horizon 637 LSO, Aspen Medical Products Inc., Irvine, CA, USA) (minimum one hour daily) and physical therapy for 12 weeks.

### 2.2. Outcome Assessment and Statistical Analysis

Our primary outcome of interest was changes in pain. Pain was evaluated using the 11-point Visual Analogue Scale (VAS) where a score of 0 indicates no pain and 10 signifies the greatest pain possible. VAS scores were collected up to 12 months. Our secondary outcome of interest was changes in function. Function was assessed using the Oswestry Disability Index (ODI). The total ODI score ranges from 0 to 50 with a higher score signifying greater disability (i.e., reduced function). The ODI comprises 10 sub-sections for different activities of daily living which include pain intensity, personal care, lifting, walking, sitting, standing, sleeping, sex life, social life, and traveling. Each sub-section score ranges from 0 to 5 with a score of 5 indicating the greatest disability level. Total ODI and sub-section scores were collected up to three months. We used those outcome measures as they were the available outcomes measured routinely in our practice.

Statistical analysis for outcomes of interest was completed using the SPSS 22 software. Data was normally distributed. A paired *t*-test was conducted to compare changes from baseline to follow-up and a *p*-value less than or equal to 0.05 indicated significance.

## 3. Results

A total of 199 patients treated between 1 January 2021 and 30 June 2023 were included in our study, comprising 85 males and 114 females (Table 1). Patients were an average age of 64.65 +/− 14.09 years and BMI of 31.30 +/− 6.47 kg/m^2^. The most common primary treatment indication for patients was lumbar radiculopathy (*n* = 139). Many patients also had one or more secondary indications. A complete list of primary and secondary treatment indications is reported in Table 2.

### 3.1. Pain

VAS scores were reported by 198 patients and reduced from 6.28 +/− 2.32 at baseline to 3.96 +/− 2.66 at three months (*p* < 0.001) (Table 3). Eighty-five patients reported their VAS score at six months, and the average was 3.74 +/− 2.73 (*p* < 0.001). At twelve months, the average VAS score was 3.23 +/− 2.29 (*p* < 0.001) for 31 patients at the follow-up.

### 3.2. Function

Function was assessed at baseline and three months. The total ODI score was collected for 199 patients. The average total ODI score reduced from 46.56 +/− 15.30 to 33.13 +/− 19.99 (*p* < 0.001) (Table 4). Scores for the categories of pain intensity, personal care, lifting, and standing on the ODI were available from 195 patients, and all were significantly improved with therapy (*p* < 0.001). One hundred and ninety-two patients reported ODI scores for sitting, where the average reduced from 1.47 +/− 1.26 to 1.11 +/− 1.14 (*p* < 0.001). Scores for ODI walking, sleeping, and sex life were obtained from 194 patients, and all were significantly improved with therapy (*p* < 0.001). The social life score improved from 2.04 +/− 1.22 to 1.52 +/−1.36 (*p* < 0.001), while the traveling score changed from 1.70 +/− 1.09 to 1.30 +/− 1.05 (*p* < 0.001).

## 4. Discussion

Our study found that the use of lumbar back bracing with physical therapy resulted in significant improvements in pain and function for patients with various CLBP conditions. VAS scores improved and decreased by 37% at three months, 42% at six months, and 48% at twelve months. The total ODI score improved by 29% at three months. Of the various domains assessed by the ODI, pain intensity and sex life had the greatest magnitude of improvement. The large improvement in the pain intensity category on the ODI supports the improvement in VAS scores.

The beneficial impact of lumbar back bracing has been evidenced in previously published studies. Morrisette et al. reported results for patients who used bracing in combination with physical therapy and found that they experienced 4.7 times higher odds of achieving 50% or greater improvement in ODI scores compared to those assigned to physical therapy alone [18]. A systematic review by Schott et al. also concluded that bracing significantly improved LBP intensity and function, with patients reporting satisfaction with the treatment modality [10]. No side effects were reported in this review.

The positive impact of lumbar orthoses on CLBP may be rooted in the modulation of central and peripheral nervous systems. One method of symptom improvement is enhanced proprioception. Bracing can stimulate mechanoreceptors on the skin and increase afferent sensory input [19]. The increase in afferent sensory activity may also reduce pain based on the Gate Control Theory of Pain [20].

Motor cortex anatomical and functional changes are also associated with chronic and recurrent low back pain. Reorganization of trunk muscle representation at the motor cortex has been associated with deficits in postural control [29]. Patients who use back stability devices have been evidenced through fMRI studies to have altered activity in areas of the brain, including the anterior cingulate cortex and cerebellum, that indicate greater confidence of stability and movement [19]. Also, pathological neuroplastic alterations in the motor cortex can be reversed by physical interventions that reduce low back pain [30].

Another proposed mechanism of action of lumbar bracing is enhanced trunk stiffness. LSO bracing has demonstrated an increase in trunk stiffness of 14% in response to trunk perturbations [18]. This mechanism of LSO function could be especially effective for patients presenting with multifidus dysfunction, functional instability, and aberrant spinal motion [16]. The prevalence of multifidus atrophy associated with chronic low back pain is 81% and could, therefore, explain the efficacy of augmented trunk stiffness via LSO treatment [28].

Risks of long-term LSO bracing have been proposed and include muscle weakness, atrophy, joint contracture, and dependence on the brace to perform physical activities [17,21]. However, a recent RCT reports no deleterious effect on trunk core musculature with continuous LSO brace use in CLBP [17]. Most recently, a systematic review by Freeman et al. found that continuous use of orthoses does not lead to muscle weakness, loss of muscle, or reduced motor performance and function [16]. It is known that joint contracture can occur from immobility [22]; however, the design of non-rigid lumbar back braces does not result in complete osteokinematic restriction. The lack of complete immobilization with non-rigid back bracing is likely the result of non-rigid brace materials and/or the inability of the brace to fully embrace the joint, thereby allowing residual joint movement [33]. Also, the wear schedule protocol is PRN for pain control. The protocol will, therefore, allow frequent mobility in the tissues and muscles while giving patients an option for brace use when their symptoms are exacerbated. Additionally, many other treatment modalities, specifically pharmacological treatments, carry the risk of adverse events when used long term. Long-term usage of NSAIDs can lead to gastrointestinal, renal, and cardiovascular problems [23]. Chronic use of acetaminophen presents similar adverse events as NSAIDs and may also contribute to the development or exacerbation of asthma [24].

The use of lumbar back bracing has also shown reduced opioid utilization in subacute low back pain [32]. The risks associated with opioids (e.g., dependence, abuse) are well recognized; however, they remain frequently used. Back pain is the most common reason for opioid prescriptions [25]. Furthermore, opioids are the most common type of drug prescribed for back pain. This is despite the fact that long-term opioid usage is not linked to greater improvements in pain and function than other non-opioid treatments [26]. It is also concerning that a significant proportion of those with opioid use disorder have CLBP [27]. Orthoses represent a noninvasive treatment option that may provide satisfactory relief for patients that is enough to shift away from reliance on opioids.

Limitations of our study included the uneven distribution of treatment indications and several patients who presented with multiple indications. This limited us to aggregate data from patients for all CLBP treatment indications. With this data aggregation, we were unable to compare outcomes for different indications. Another limitation was the retrospective study design. This limitation resulted in our inability to retrieve longer-term data regarding ODI and the loss of a large number of patients at the 6- and 12-month follow-ups for VAS scores. Future studies should include patients with a single treatment indication or a more even distribution of treatment indications and evaluate them for a longer period of time (>12 months) in order to determine long-term safety and efficacy. Additionally, it would be of interest to assess the use of bracing for patients with nonspecific CLBP since they comprise the largest proportion of the CLBP population. Lastly, when examining bracing in combination with physical therapy, the effect of physical therapy alone should be evaluated to determine the extent of additional benefit that bracing may provide.

## 5. Limitations

This was a retrospective study, and we had to exclude patients with incomplete follow-up data. We did not have a control group, did not address differences in outcomes among patients with different low back disease conditions, and did not have long-term follow-up.

## 6. Conclusions

Our study suggests that the use of back bracing in combination with physical therapy results in significant improvements in pain and function. While there may not be enough evidence to consider bracing as a standalone treatment, it should be recognized as an effective PRN adjunctive therapy.

## Figures and Tables

**Table 1:** Patient demographics.

Sex	Frequency	Male—85 Female—114
Age	Mean (SD) [Range]	64.65 years (14.09) (24–96) *n* = 199
Weight		200.26 lbs (48.58) (97–450) *n* = 157
BMI		31.30 kg/m^2^ (6.47) (17.83–56.25) *n* = 155

**Table 2:** Treatment indications.

Primary Indication *	Secondary Indication(s) * (97–450)
Radiculopathy, lumbar—139	Lumbago with sciatica, right side—1 Vertebrogenic low back pain—1 Segmental and somatic dysfunction of lumbar region—2 Radiculopathy, Lumbosacral region—1 Spinal stenosis lumbar region without neurogenic claudication—1 Spinal stenosis, lumbosacral region—1 Other intervertebral disc displacement, lumbar region—1 Other specified dorsopathies lumbar region—1 Other specified dorsopathies lumbar region + Segmental and somatic dysfunction of lumbar region—1 Other intervertebral disc degeneration, lumbar region—2 Other intervertebral disc degeneration, lumbar region + Segmental and somatic dysfunction of lumbar region + Spinal stenosis, lumbosacral region + Other spondylosis, lumbar region—1 Other intervertebral disc degeneration, lumbar region + Subluxation complex (vertebral) of lumbar region—1 Other intervertebral disc degeneration, lumbar region + Spondylolisthesis, lumbar region—1 Other low back pain + Intervertebral disc disorders with radiculopathy, lumbar region—1 Intervertebral disc disorders with radiculopathy, lumbar region—1 Other spondylosis, lumbar region—1 Other spondylosis, sacral, sacrococcygeal region—1 Spinal stenosis, lumbar region with neurogenic claudication + Spondylosis without myelopathy or radiculopathy, lumbosacral region + Spinal stenosis, site unspecified—1 Spinal stenosis, lumbar region with neurogenic claudication + Fusion of spine, lumbar region—1 Lumbago with sciatica, unspecified side—2 Spondylosis without myelopathy or radiculopathy, lumbosacral region—1 Vertebrogenic low back pain—2
Segmental and somatic dysfunction of lumbar region—8	Radiculopathy, Lumbar—2 Radiculopathy, Lumbar + Vertebrogenic low back pain—1 Other specified dorsopathies lumbar region—1 Other specified dorsopathies lumbar region + Segmental and somatic dysfunction of pelvic region + Other spondylosis, sacral, sacrococcygeal region + Radiculopathy, Lumbar—1 Other intervertebral disc degeneration, lumbar region + Spinal instabilities, lumbar region—1
Radiculopathy, Lumbosacral region—2	Segmental and somatic dysfunction of lumbar region—1
Spinal stenosis thoracolumbar region—1	N/A
Spinal stenosis lumbar region without neurogenic claudication—4	N/A
Spinal stenosis, lumbosacral region—2	Other intervertebral disc degeneration, lumbar region—1 Other intervertebral disc degeneration, lumbar region + Other spondylosis, lumbar region—1
Other specified dorsopathies lumbar region—1	Segmental and somatic dysfunction of lumbar region + Other intervertebral disc displacement, lumbar region—1
Low back pain—10	N/A
Other intervertebral disc degeneration, lumbar region—1	N/A
Other low back pain—7	N/A
Lumbago with sciatica L side—2	N/A
Spinal instabilities, lumbar region—2	Other intervertebral disc degeneration, lumbar region—1
Spondylolisthesis, lumbar region—1	N/A
Lumbago with sciatica, right side—2	N/A
Other spondylosis with myelopathy, lumbar region—1	Spinal stenosis, lumbosacral region—1
Sciatica L side—1	N/A
Other spondylosis, sacral, sacrococcygeal region—2	Segmental and somatic dysfunction of pelvic region + Segmental and somatic dysfunction of lumbar region + Radiculopathy, Lumbar—1 Segmental and somatic dysfunction of pelvic region + Other specified dorsopathies lumbar region + Segmental and somatic dysfunction of lumbar region—1
Other spondylosis with radiculopathy, lumbar region—1	Other intervertebral disc degeneration, lumbar region + Segmental and somatic dysfunction of lumbar region + Radiculopathy, Lumbar—1
Spinal stenosis, lumbar region with neurogenic claudication—6	Radiculopathy, Lumbar—1
Lumbago with sciatica, unspecified side—4	Radiculopathy, Lumbar—1 Radiculopathy, Lumbosacral region—1 Other intervertebral disc degeneration, lumbar region—1
Spinal stenosis, site unspecified—1	N/A
Vertebrogenic low back pain—1	N/A

* Based on ICD-10 codes.

**Table 3:** VAS outcome summary.

VAS	Mean (SD)	Sample Size	*p*-Value
Baseline	6.28 (2.32)	198	*p* < 0.001
3 months	3.96 (2.66)		
Baseline	6.47 (2.29)	85	*p* < 0.001
6 months	3.74 (2.73)		
Baseline	6.16 (1.72)	31	*p* < 0.001
12 months	3.23 (2.29)		

**Table 4:** ODI outcomes.

	Mean (SD)	Sample Size	*p*-Value
Total ODI score			
Baseline	46.56 (15.30)	199	*p* < 0.001
3 months	33.13 (19.99)		
Pain intensity			
Baseline	3.10 (1.25)	195	*p* < 0.001
3 months	1.98 (1.54)		
Personal care			
Baseline	1.53 (1.11)	195	*p* < 0.001
3 months	0.97 (1.05)		
Lifting			
Baseline	2.85 (1.69)	195	*p* < 0.001
3 months	2.16 (1.75)		
Standing			
Baseline	2.88 (1.31)	195	*p* < 0.001
3 months	2.25 (1.48)		
Sitting			
Baseline	1.47 (1.26)	192	*p* < 0.001
3 months	1.11 (1.14)		
Walking			
Baseline	2.97 (1.64)	194	*p* < 0.001
3 months	2.27 (1.75)		
Sleeping			
Baseline	1.73 (1.23)	194	*p* < 0.001
3 months	1.16 (1.13)		
Sex life			
Baseline	2.92 (1.12)	194	*p* < 0.001
3 months	1.62 (1.21)		
Social life			
Baseline	2.04 (1.22)	191	*p* < 0.001
3 months	1.52 (1.36)		
Traveling			
Baseline	1.70 (1.09)	191	*p* < 0.001
3 months	1.30 (1.05)		

## Data Availability

For HIPPA reasons we can not share the data in public.
